# A Tiered Analytical Approach for Investigating Poor Quality Emergency Contraceptives

**DOI:** 10.1371/journal.pone.0095353

**Published:** 2014-04-18

**Authors:** María Eugenia Monge, Prabha Dwivedi, Manshui Zhou, Michael Payne, Chris Harris, Blaine House, Yvonne Juggins, Peter Cizmarik, Paul N. Newton, Facundo M. Fernández, David Jenkins

**Affiliations:** 1 School of Chemistry and Biochemistry, Georgia Institute of Technology, Atlanta, Georgia, United States of America; 2 Product Quality and Compliance, FHI 360, Durham, North Carolina, United States of America; 3 Lao-Oxford-Mahosot Hospital-Wellcome Trust Research Unit, Microbiology Laboratory, Mahosot Hospital, Vientiane, Lao PDR; 4 Centre for Tropical Medicine, Churchill Hospital, University of Oxford, Oxford, United Kingdom; 5 WorldWide Antimalarial Resistance Network, Churchill Hospital, University of Oxford, Oxford, United Kingdom; University of Nevada School of Medicine, United States of America

## Abstract

Reproductive health has been deleteriously affected by poor quality medicines. Emergency contraceptive pills (ECPs) are an important birth control method that women can use after unprotected coitus for reducing the risk of pregnancy. In response to the detection of poor quality ECPs commercially available in the Peruvian market we developed a tiered multi-platform analytical strategy. In a survey to assess ECP medicine quality in Peru, 7 out of 25 different batches showed inadequate release of levonorgestrel by dissolution testing or improper amounts of active ingredient. One batch was found to contain a wrong active ingredient, with no detectable levonorgestrel. By combining ultrahigh performance liquid chromatography-ion mobility spectrometry-mass spectrometry (UHPLC-IMS-MS) and direct analysis in real time MS (DART-MS) the unknown compound was identified as the antibiotic sulfamethoxazole. Quantitation by UHPLC-triple quadrupole tandem MS (QqQ-MS/MS) indicated that the wrong ingredient was present in the ECP sample at levels which could have significant physiological effects. Further chemical characterization of the poor quality ECP samples included the identification of the excipients by 2D Diffusion-Ordered Nuclear Magnetic Resonance Spectroscopy (DOSY ^1^H NMR) indicating the presence of lactose and magnesium stearate.

## Introduction

Over the last decade, there has been increasing interest and concern regarding the quality of medicines worldwide, stimulating research into their epidemiology and novel detection methods. Much of this work has focused on antimalarials [1], [2] but there is evidence that poor quality antibiotics and anti-tuberculosis drugs are focally common. Recent cases of poor quality medicines include contaminated isosorbide mononitrate, which led to 125 deaths in Pakistan [3]; anti-visceral leishmaniasis medications found in Bangladesh that did not contain any of the expected active pharmaceutical ingredient (API) miltefosine [4]; and contaminated methylprednisolone injections that led to an epidemic of fungal infections in the US [5]. Poor quality medicines have a wide range of public health impacts, ranging from poor outcomes, including treatment failure and even death; economic consequences for patients, for the society, and the legitimate pharmaceutical industry; and engendering drug resistance. Therefore, assessing the prevalence of poor quality drugs is an essential step in protecting the welfare of patients.

There are three main categories of poor quality medicines: i) substandard medicines, resulting from negligent or unintentional factory error; ii) falsified medicines, which arise from fraudulent, criminal production; and iii) degraded medicines, which result from deterioration after factory production such as poor storage conditions; *e.g.* heat, humidity, etc. Because of intellectual property connotations, the term counterfeit is being replaced with the term falsified that is devoid of such considerations, although the precise definition of these terms has yet to be agreed globally [6], [7]. Falsified drugs may not contain the active ingredient, may contain wrong ingredients or may even contain toxic compounds. Substandard medicines usually contain less than the stated active ingredient [8].

There are diverse techniques to analyze pharmaceuticals and identify poor quality medicines [9], [10]. Typically, visual inspection of product and packaging is performed first to identify salient differences with the genuine product; this is followed by tests for physical properties such as disintegration, reflectance spectroscopy, or refractive index; and chemical tests that include colorimetry, dissolution, thin layer chromatography (TLC) and liquid chromatography with spectrophotometric detection. The investigation of poor quality medicines that lack the expected API but may instead contain a different compound requires the use of near infrared (NIR), Raman, UV-visible spectroscopy, or even more sophisticated analytical techniques such as nuclear magnetic resonance spectroscopy (NMR) or mass spectrometry (MS).

Reproductive health has been affected by poor quality health products such as falsified hormonal contraceptives and poor quality condoms [11]. In 1998, counterfeit contraceptive pills were reported in Brazil [12]; in 2004, the US FDA warned the public about an internet site selling counterfeit contraceptive transdermal patches [13]. More recently, the US FDA issued an alert of a potentially-ineffective and suspect falsified emergency contraceptive labeled as Evital [14], and the World Health Organization (WHO) warned of falsified ‘Postinor 2’ (levonorgestrel 0.75 mg) in Nigeria (150,000 doses), Ghana, Kenya and Angola, containing no detectable levonorgestrel [15].

Emergency contraceptive pills (ECPs) are an important birth control method that women can use after unprotected coitus [16]. In the US, more than 5 million women aged 15–44 years used emergency contraception between 2006 and 2010 [17]. There are over 150 different brands of ECPs containing levonorgestrel, mifepristone, or ulipristal acetate available globally, which are manufactured in over 25 countries [18]. In South American countries, a wide variety of registered ECP brands exists. Peru has over 20 brands in the market that are manufactured in 10 different countries [18]. With the high number of ECP brands and source countries, this may increase the risk of substandard, degraded or falsified products. The unwitting use of poor quality ECPs may result in unwanted pregnancy, and diverse medical and social consequences.

This work presents the first report on a multi-platform analytical strategy used to investigate suspect poor quality ECPs found through a small survey conducted in the Peruvian market.

## Materials and Methods

### Sample collection

Samples of levonorgestrel tablets (0.75 mg and 1.5 mg) were purchased at 15 pharmacies and pharmaceutical distributors in Lima, Peru, using a convenience sampling method [9]. At least 60 tablets were purchased per sample, consisting of the same batch number for a given brand. For each sample, the brand name, manufacturer, lot number, number of tablets/blister packs purchased, manufacturing date, expiration date, date purchased, purchaser and address of purchase were recorded. Tablets were collected from 25 different product batches encompassing 20 brands labeled as manufactured in nine countries (Argentina, Chile, China, Colombia, Hungary, India, Pakistan, Peru, and Uruguay). Photographs were taken of the front and back of the outer packaging and blister packs. Results are reported, as far as possible, according to the Medicine Quality Assessment Reporting Guidelines (MEDQUARG) [19].

### Chemicals

Omnisolv LC-MS grade acetonitrile and hydrochloric acid 37% were purchased from EMD (Billerica, MA, USA). LC-MS grade methanol was purchased from J.T. Baker Avantor Performance Materials, Inc. (Center Valley, PA, USA). HPLC grade cyclohexane and acetone were purchased from Alfa Aesar (Ward Hill, MA, USA); HPLC grade acetonitrile and methanol were purchased from Honeywell Burdick & Jackson (Morristown, NJ, USA). Ethanol (190 proof) was obtained from Decon Labs, Inc. (King of Prussia, PA, USA). Ultrapure water, with 18.2 MΩ cm resistivity (Hydro Pico Pure system and Barnstead Nanopure UV ultrapure water system, USA) was used to prepare solutions and mobile phases. Levonorgestrel (99.9%) and ethinyl estradiol (99.9%) were purchased through the EDQM/WHO International Chemical Reference Substances program, and norgestrel (99.7%) was purchased from the US Pharmacopeia. Sulfamethoxazole standard (ACS reagent ≥99%), methanol-d_4_ (CD_4_O, 99.8 atom% D), and deuterium oxide (D_2_O 99.9 atom% D) were purchased from Sigma-Aldrich Co. (St. Louis, MO, USA). Ammonium acetate (HPLC ≥99.0%) was purchased from Fluka Chemical Corp. (Milwaukee, WI, USA). Sodium dodecyl sulfate was purchased from BioRad (Hercules, CA, USA); γ-cyclodextrin (γ-CD) was purchased from Wacker (München, Germany). PTFE syringe filters, 0.45 µm were purchased from VWR (Radnor, PA, USA) and Silica gel 60 20×20 cm, 250 µm thickness TLC plates were purchased from EMD (Billerica, MA, USA).

### Thin Layer Chromatography (TLC) analysis [20]

TLC analysis was conducted with 10 µL applications of standards and samples using a mobile phase of cyclohexane:acetone 70∶30 v/v with observation using 254 nm light. Samples were prepared by dissolving a mass of powdered tablets equivalent to 1.5 mg of levonorgestrel in 5 mL of acetonitrile, followed by filtration. Standards of 0.3 mg mL^−1^ levonorgestrel RS were prepared in acetonitrile.

### High Performance Liquid Chromatography with Diode Array Detection (HPLC-DAD)

HPLC-DAD was used to determine the limit of dextronorgestrel, related substances, to perform the content uniformity assay, and analyze standards and samples from dissolution tests, following the International Pharmacopeia specifications for levonorgestrel tablets [20]. HPLC-DAD analysis was conducted with an Agilent HP1200 consisting of a G1329A autosampler, a G1322A degasser, a G1311A quaternary pump, a G1316A column thermostat, and a G1315B diode array detector (Agilent Technologies, Santa Clara, CA, USA). Data was acquired using ChemStation software (Agilent Technologies). Specific details for each assay are provided below.

#### Limit of Dextronorgestrel

Chiral HPLC was utilized to quantify the level of dextronorgestrel, while verifying the presence of levonorgestrel. HPLC was conducted on a Hypersil ODS 2 C_18_ column (15 cm×4.6 mm, 5 µm particle size, Waters Corporation, Milford, MA, USA). The chromatographic method used an isocratic mobile phase of methanol: water with 1% (w/w) γ-CD 50∶50 v/v. The flow rate was 1.5 mL min^-1^ and the injection volume was 20 µL. The detection was performed by monitoring absorbance at 242 nm. For preparing test samples with 0.12 mg mL^−1^ of levonorgestrel (D1), an amount of powdered tablet (equivalent to 3 mg of levonorgestrel) was transferred to a 25.0 mL volumetric flask with 15 mL of methanol:water 80∶20 v/v (diluent), heated at 60°C for 10 minutes (with periodic shaking), brought to volume with diluent, and filtered through a 0.45-µm filter. Test samples D1were then diluted to reach 6 µg mL^−1^ of levonorgestrel (D2). A norgestrel standard (N) was prepared at 12 µg mL^−1^ norgestrel RS in diluent. A levonorgestrel standard (L1) was prepared at 6 µg mL^−1^ levonorgestrel RS in diluent, and diluted to 0.12 µg mL^−1^ in diluent (L2). Standard N was used to designate the retention time for dextronorgestrel and levonorgestrel, requiring a resolution of at least 1.5. Identification of levonorgestrel was conducted through retention time matching between the standard L1 and test sample D2. Quantification of dextronorgestrel was obtained from injections of standard L2 and test sample D1. To yield full compliance with the International Pharmacopeia specifications [20] for the limit of dextronorgestrel, the chromatographic peak area for dextronorgestrel in D1 should not be greater than that of levonorgestrel in L2 at 0.1%.

#### Related Substances

Samples were analyzed by HPLC-DAD using a Spherisorb ODS 2 C_18_ column (25 cm×4.6 mm, 5 µm particle size, Waters Corporation, Milford, MA, USA). An isocratic mobile phase of methanol:acetonitrile:water 15∶35∶50 v/v was used in the chromatographic method with a flow rate of 1.2 mL min^−1^. The column temperature was set at 30°C, and the volume of injection was 100 µL. The detection was performed by monitoring absorbance at 220 nm. Test samples (RS1) were prepared by transferring a quantity of powdered tablets equivalent to 0.18 mg of levonorgestrel in 5 mL of diluent (50% v/v methanol in water). These were sonicated for 30 minutes, stirred for 15 minutes, centrifuged (4000 RPM, 10 min), and the supernatant was separated for analysis. Test samples (RS2) were prepared by diluting RS1 to 0.36 µg mL^−1^ of levonorgestrel in diluent. A standard of 4 µg mL^−1^ ethinyl estradiol and levonorgestrel was injected to ensure that a resolution of at least 12 could be obtained between the two compounds. With injections of RS1 and RS2, the area of any peak from RS1 (other than levonorgestrel) should not be greater than the area of levonorgestrel in RS2 (or greater than a level of 1.0% relative to levonorgestrel). Also from RS1, the sum of areas of all peaks (other than levonorgestrel) should not be greater than twice the area of levonorgestrel in RS2 (or greater than a level of 2.0% relative to levonorgestrel).

#### Assay/Content Uniformity

Samples were analyzed with HPLC using a Spherisorb ODS 2 C_18_ column (15 cm×4.6 mm, 5 µm particle size, Waters Corporation, Milford, MA, USA). An isocratic mobile phase of acetonitrile:water 50∶50 v/v was used in this chromatographic method with a flow rate of 1.3 mL min^−1^. The column was held at ambient temperature, the volume of injection was 25 µL, and the detection was at 220 nm. Test samples were prepared by transferring one powdered tablet to an enclosed test tube with 5.0 mL of mobile phase. The suspension was sonicated for 45 minutes, shaken for 15 minutes, and centrifuged (4000 RPM, 10 min). The supernatant was further diluted to a theoretical concentration of 6 µg mL^−1^ of levonorgestrel. Standards of 6 µg mL^−1^ levonorgestrel RS prepared in mobile phase were used to determine the amount of levonorgestrel in test sample injections. Assay and content uniformity were judged from the % label claim values of API found for individual tablets. Unless indicated otherwise, 10 tablets were analyzed per test sample. The assay requires the average to be within 90.0%–110.0% of the label claim. For content uniformity, each of 10 tablets should contain within ±15% of the average amount. If one individual tablet deviates by more than ±15% but is within ±25% of the average amount, another group of 20 tablets are tested. No more than one tablet out of 30 can deviate by more than ±15% of the average amount and none should deviate by more than ±25% of the average amount.

#### Dissolution Test

Analysis was conducted with a VK 7000 Varian dissolution apparatus (VanKel Technology Group, Cary, NC, USA) with paddles rotating at 75 rpm in 500 mL of medium comprised of 0.1% sodium dodecyl sulfate in a 0.1 M HCl aqueous solution. A sample aliquot of 10 mL was centrifuged (4000 RPM, 10 min) 30 min after dropping the individual tablets in the vessels, and the supernatant was used for analysis. Standards were prepared by dissolving 30 mg of levonorgestrel RS in 50 mL of ethanol via sonication, and further dilution in dissolution medium to a final concentration of 6 µg mL^−1^. Standards and test samples were analyzed under the same HPLC conditions utilized for the assay/content uniformity. The testing protocol was conducted in a series of stages, proceeding to the next stage only if a given stage was non-compliant. Tablets were evaluated based on the percentage of released API relative to the theoretical amount in the tablet. The criteria utilized for each stage in the testing protocol was as follows: Stage 1 =  individual values of 6 tablets must be greater than or equal to 80%, Stage 2 =  mean value of 12 tablets must be greater than or equal to 75%, and no tablets can be less than 60%, Stage 3 =  mean value of 24 tablets must be greater than or equal to 75%, no more than 2 tablets can be less than 60%, and no tablet can be less than 50%.

### Direct Analysis in Real Time-Mass Spectrometry and Tandem Mass Spectrometry (DART-MS and DART MS/MS)

MS screening was performed with a commercial DART-100 ion source (IonSense, Inc., Saugus, MA, USA) coupled to a quadrupole-time of flight (Q-TOF) mass spectrometer (Bruker micrOTOF-Q I; Bremen, Germany) through a VAPUR interface. The VAPUR interface was designed to accommodate for the gas flow of the DART source and consists of a custom gas-ion separator tube (GIST; IonSense Inc. Saugus, MA, USA) connected to a Vacuubrand 2 C diaphragm pump (Vacuubrand, Wertheim, Germany), the outlet of which is connected to an exhaust line. The ion source exit, interface inlet and mass spectrometer inlet are aligned along the same axis. Ions created between the ion source exit and the interface inlet (ionization region), are dragged into the mass spectrometer by the suction created by the diaphragm pump and the vacuum system of the mass spectrometer. By partially removing unionized molecules and plasma gases from the dragged plume, the interface also serves to reduce the vacuum load on the first differentially pumped region of the mass spectrometer and maintain the instrument's pressure requirements for operation. The DART ion source was operated with helium (UHP 99.999%, Airgas, Atlanta, GA, USA) at a 1 L min^−1^ flow rate and at 150°C gas temperature. Potentials of −3500 V, +200 V, and +150 V were applied at the needle electrode, discharge grid, and exit grid of the ion source, respectively. The mass spectrometer settings were as follows: end plate offset −500 V, capillary −2000 V, dry gas (nitrogen) 1.2 L min^−1^, dry gas temperature 150°C, spectra acquired at 1 Hz in the 50–1200 *m/z* range. A methanol solution of a 50∶50 mixture of polyethylene glycol 400 and 600 (PEG, Sigma Aldrich, St. Louis, MO, USA) was used as the mass calibration standard. The mass spectrometer provided a mass resolving power of ∼12,000 m/Δ*m* at *m/z* 393.2095 and ∼9000 m/Δ*m* at *m/z* 151.0964, and a typical mass accuracy of 2–5 ppm was obtained for acetaminophen as test compound. All experiments were performed in positive-ion detection mode and all samples were analyzed under identical instrumental and experimental conditions. For analysis, a few particles of each tablet were deposited on a glass capillary by rubbing the tablet dust on the capillary. Two different tablets from sample 2a and levonorgestrel-containing ECPs as well as standards were analyzed in technical triplicates. The capillary with the deposited sample was introduced in front of the plasma plume exiting the DART source for sample ionization and subsequent detection by the mass spectrometer. Tandem MS collision induced dissociation experiments were performed to confirm the identity of analyte peaks detected in the mass spectrometer. High purity nitrogen (99.998%) was used as the collision gas. Mass spectral data processing was performed using Bruker Daltonics Data Analysis Version 4.0 software.

### Ultra High Performance Liquid Chromatography-Ion Mobility Spectrometry-Mass Spectrometry and Tandem Mass Spectrometry (UHPLC-IMS-MS, UHPLC-MS/MS)

A measured mass of each tablet was dissolved in 15 mL of LC-MS grade methanol, and sonicated for 30 min, 5 mL of nanopure water were added to help solubilization, and methanol was finally added to complete 25.0 mL final volume. The sample was vortex-mixed for 2 min, centrifuged (3 min, 13,000×*g*), and the supernatant was then 10-fold diluted for UHPLC-IMS-MS and UHPLC-MS/MS analysis. Standards of levonorgestrel and sulfamethoxazole were prepared in methanol:water 80∶20 v/v.

UHPLC-IMS-MS analysis was performed using a Waters ACQUITY Ultra Performance LC (Waters Corporation, Manchester, UK) system, coupled to a Synapt G2 High Definition Mass Spectrometry system (Waters Corporation, Manchester, UK), which is a hybrid quadrupole-ion mobility-orthogonal acceleration time-of-flight instrument, with typical resolving power of 20,000 m/Δ*m* (FWHM) and mass accuracy of 9 ppm at *m/z* 556.2771. The UHPLC system was fitted with a Waters ACQUITY UPLC BEH C_18_ column (1.0×100 mm, 1.7 µm particle size, Waters Corporation, Milford, MA, USA). The optimized traveling wave ion mobility spectrometry (TWIMS) conditions were as follows: wave height ramped between 8 and 20 V; wave velocity ramped between 300 and 600 m s^−1^; IMS gas flow rate: 90 mL min^−1^; helium gas flow rate: 180 mL min^−1^. The instrument was operated in positive ion mode with a probe capillary voltage of 3 kV, and a sampling cone voltage of 45 V. The source and desolvation temperatures were set to 120°C and 250°C, respectively; and the nitrogen desolvation flow rate was set to 650 L h^−1^. The mass spectrometer was calibrated across the 50–1200 *m/z* range using a 0.5 mM sodium formate solution prepared in 90∶10 2-propanol:water v/v. Data were mass corrected during acquisition using a leucine encephalin (*m/z* 554.2615) reference spray (LockSpray) infused at 2 µL min^−1^. The scan time was set to 1 s. Data acquisition and processing was carried out using MassLynx v4.1 and Drift Scope v2.1 (Waters Corp.). The chromatographic method involved elution with acetonitrile (mobile phase A) and water with 0.1% acetic acid (mobile phase B) using the following gradient duration program: 0–4 min 10% A; 4–5 min 10–100% A; 5–5.4 min 100–10% A; 5.4–6 min 10% A. The flow rate was 0.3 mL min^−1^, and the column temperature was 45°C. The autosampler tray temperature was set to 5°C, the injection volume was 5 µL and the total run time was set to 6 min. Two different tablets from sample 2a and standards were analyzed in technical triplicates.

UHPLC-MS/MS analysis was performed with a 1290 Infinity UHPLC unit coupled to a 6430 Triple Quadrupole LC-MS system (both Agilent Technologies, Santa Clara, CA, USA) using an identical column and chromatographic conditions as those described above for UHPLC-IMS-MS analysis. Ultrahigh purity nitrogen (≥99.999%) was used as the collision gas. The Q1 and Q3 quadrupoles were set to unit resolution. Data acquisition and processing were carried out using Mass Hunter B.04.01 software (Agilent Technologies, Santa Clara, CA, USA). For sulfamethoxazole MS/MS experiments, the instrument was operated in positive electrospray ion mode with a capillary voltage of 4 kV. The nebulizer pressure was set to 25 psi; the desolvation gas temperature to 350°C, and the nitrogen flow rate to 10 L min^−1^. Two different tablets from sample 2a were analyzed for quantitation of sulfamethoxazole. At least 10 points were acquired across chromatographic peaks to ensure proper quantitation. Three technical replicates for standard solutions and sample 2a were analyzed. The standard deviation (SD) of each calibration point was observed to statistically increase with concentration (two-sided F-test, p = 0.05). Therefore, weights (*w _i_ = s_i_^−2^*) were used to account for heteroscedasticity, where *s_i_* is the SD of the different technical replicates.

### ^1^H and 2D DOSY ^1^H NMR analysis

Samples were prepared by dissolving a mass of powdered tablets equivalent to 5 mg of analyte in 0.7 mL of methanol-d_4_:deuterium oxide 80∶20 v/v, followed by sonication for 30 minutes, and centrifugation (3 min, 13,000×*g*). The supernatant was poured into a 5-mm NMR tube for analysis. Individual standards of lactose, magnesium stearate, levonorgestrel, and sulfamethoxazole were prepared in the same deuterated solvent mixture reaching final concentrations of 7 mg mL^−1^. NMR experiments were performed in duplicate at a temperature of 298 K on a Bruker-Biospin Avance II 500 NMR spectrometer, operated at a ^1^H frequency of 500 MHz. The instrument was equipped with a 5 mm ^1^H/X-broadband probe with gradient in the z-direction. Acquisition parameters for the 1D ^1^H NMR experiments comprised ^1^H excitation with a 30 degree pulse followed by an acquisition of 65536 data points during a time of 3.17 s; and followed by a recycle delay of 1 s. A total of 16 scans were accumulated. Data were referenced with respect to the solvent peak of the deuterated solvent (methanol-d_4_ at 3.31 ppm). The parameters for 2D DOSY ^1^H NMR experiments were as follows: LED pulse sequence with bipolar gradients, Δ = 50 ms diffusion time, δ = 2.2 ms gradient duration; 2s repetition delay, and 16 averages. Diffusion was encoded using gradient pulses with a sinusoidal shape with 16 gradient steps ranging from 0.7 to 32 Gauss cm^−1^. All NMR data were processed using TOPSPIN software (Bruker).

## Results

Full compliance with the International Pharmacopeia specifications for levonorgestrel tablets [20] was observed for 18 of 25 ECP samples (72%, Table 1). For the seven non-compliant samples, five failed dissolution testing, with samples 2c, 3b, 4, and 6 giving dissolution results ranging from 57.8% to 73.0% (minimum acceptable limit of 75%). Sample 10 gave no release of levonorgestrel, while also exhibiting poor results for related substances (unspecified impurities) and content uniformity. In addition, samples 2c and 17 revealed poor content uniformity with a wide range of levonorgestrel levels between tablets.

**Table 1:** Summary of results for the ECP quality survey^a^.

API Level	Product Code - Expiration Date	Dissolution (Stage; AVG)^b^	Related Substances^c^	Assay-CU^d^ (Min. – Max.)
1.5 mg	1 - Feb 2016	S2; 80.1	S-0.1%, T-0.6%	100.0; 97.7–102.4
	2a - May 2015	**NC**	**NC**	**NC**
	2b - Aug 2016	S2; 77.4	S-0.3%, T-1.0%	95.1; 82.2–103.4
	2c – Dec 2016	**S3; 58.5**	S-0.4%, T-1.3%	**96.2; 85.4–136.6**
	3a - Oct 2015	S1; 94.3	S-0.4%, T-1.1%	101.3; 100.5–102.6
	3b - Dec 2016	**S3; 73.0**	S-0.6%, T-1.8%	97.6; 90.0–105.7
	4 - Oct 2014	**S1; 59.6** e	S-0.9%, T-1.5%	96.7; 93.3–98.0
	5a - Dec 2013	S1; 93.8	S-0.4%, T-1.4%	97.3; 96.5–98.6
	5b - Nov 2013	S1; 98.2	S-0.3%, T-1.4%	97.0; 88.2–99.5
	6 - Apr 2014	**S3; 57.8**	S-0.3%, T-1.3%	99.0; 92.9–102.6
	7 - Oct 2013	S1; 95.2	S-0.3%, T-1.1%	91.4; 86.3–93.8
	8 - Apr 2013	S1; 95.7	S-0.4%, T-1.8%	96.0; 93.6–97.7
	9 - May 2014	S2; 76.7	S-0.5%, T-1.4%	98.3; 94.8–101.0
0.75 mg	10 - Oct 2014	**S1; 0.0** f	**S-62.3%, T-72.9%** g	**92.3; 72.9–126.5**
	11 - Dec 2014	S2; 77.0	S-0.2%, T-0.4%	100.4; 97.7–103.7
	12 - Jul 2013	S1; 83.6	S-0.4%, T-1.2%	99.9; 95.0–105.6
	13 - Sept 2015	S2; 77.8	S-0.2%, T-0.6%	99.3; 98.3–100.7
	14a - June 2014	S1; 90.6	S-0.5%, T-1.6%	101.9; 100.1–104.7
	14b - June 2014	S1; 88.3	S-0.5%, T-1.5%	102.0; 100.8–103.6
	15 - Nov 2013	S1; 92.5	S-0.3%, T-1.3%	99.8; 93.2–105.3
	16 - Sept 2013	S1; 89.5	S-0.4%, T-0.5%	102.5; 100.3–105.6
	17 - Oct 2013	S1; 95.2	S-0.4%, T-2.0%	**96.4; 71.5–115.1** h
	18 - Aug 2013	S1; 92.9	S-0.3%, T-1.3%	98.3; 94.2–103.3
	19 - Nov 2013	S2; 79.8	S-0.3%, T-1.6%	101.7; 94.8–106.2
	20 - Mar 2015	S1; 101.5	S-0.4%, T-1.5%	104.2; 99.8–116.4

a Product Labels – number indicates a different brand, where a subsequent letter indicates a different batch. Bold text indicates non-compliance. With the exception of 2a, all samples yielded compliant results for identification (TLC and confirmation of levonorgestrel through the HPLC evaluation for dextronorgestrel). No sample showed evidence of dextronorgestrel.

b Dissolution presented with the stage required and average result.

c Related Substances – the level of any single related substance (S) should be less than 1.0% and the total amount of related substances (T) should be less than 2.0%.

d Assay and Content Uniformity results presented as the average results and the minimum / maximum result found.

e Only Stage 1 was conducted because 3 tablets were found to be below 60%.

f No active release was observed on the 6 tablets tested. Additional tablets were tested for longer periods of release (1 hour) and observed ∼2–5% release of active.

g A single peak was observed in the chromatogram (other than for levonorgestrel) which made a large contribution to the level of observed impurities.

h 30 tablets tested.

**Figure 1:**
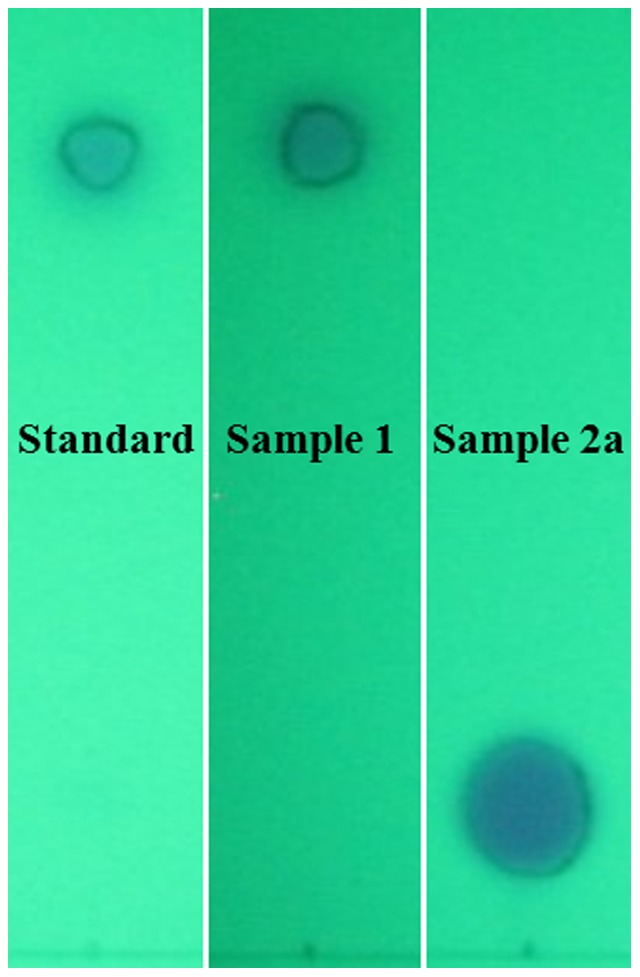
TLC analysis. Results for a levonorgestrel standard, sample 1 (positive control), and sample 2a observed under 254 nm light.

**Figure 2:**
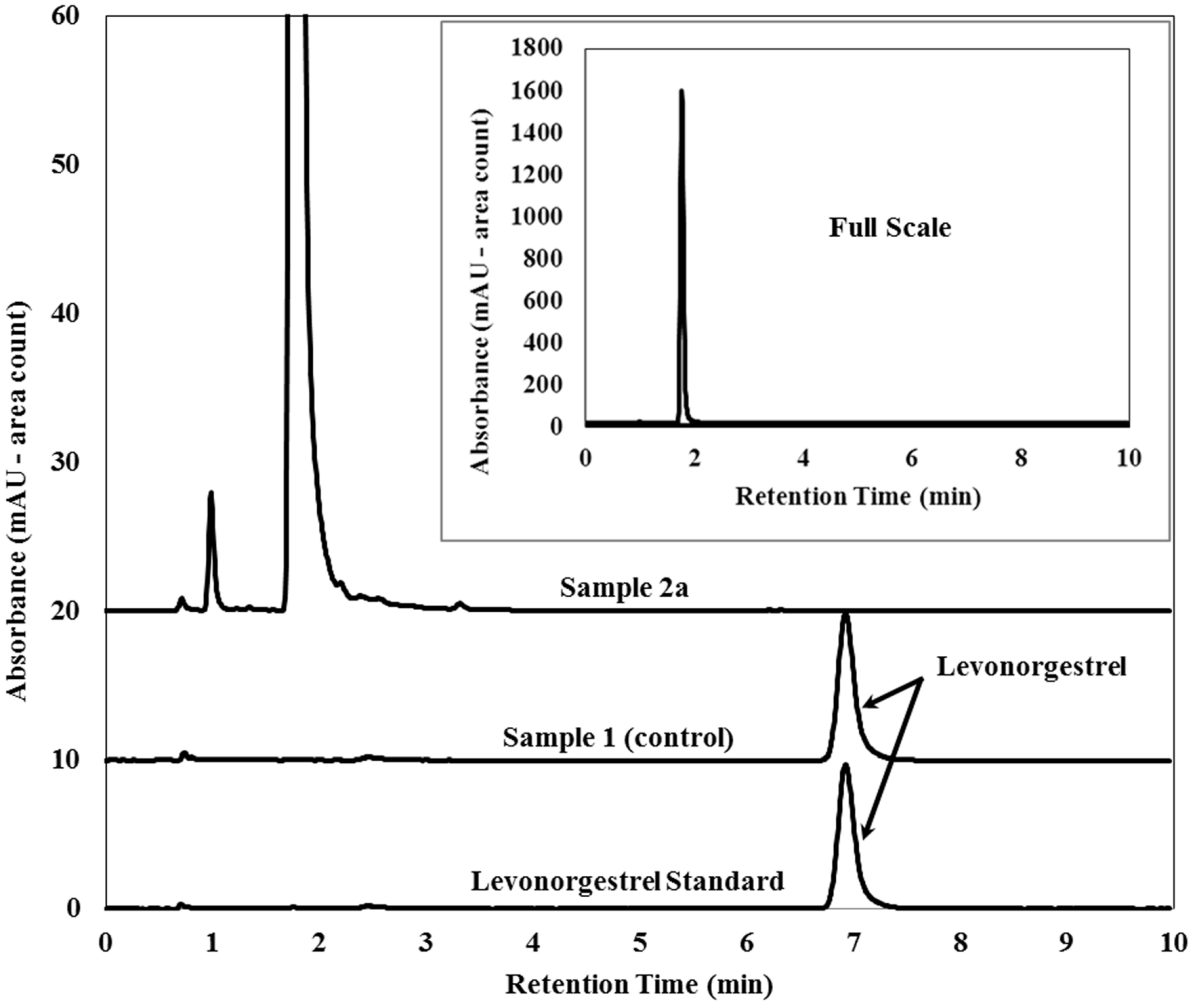
HPLC-DAD analysis. HPLC chromatograms from assay / content uniformity analysis for a levonorgestrel standard, sample 1 (positive control), and sample 2a.

**Figure 3:**
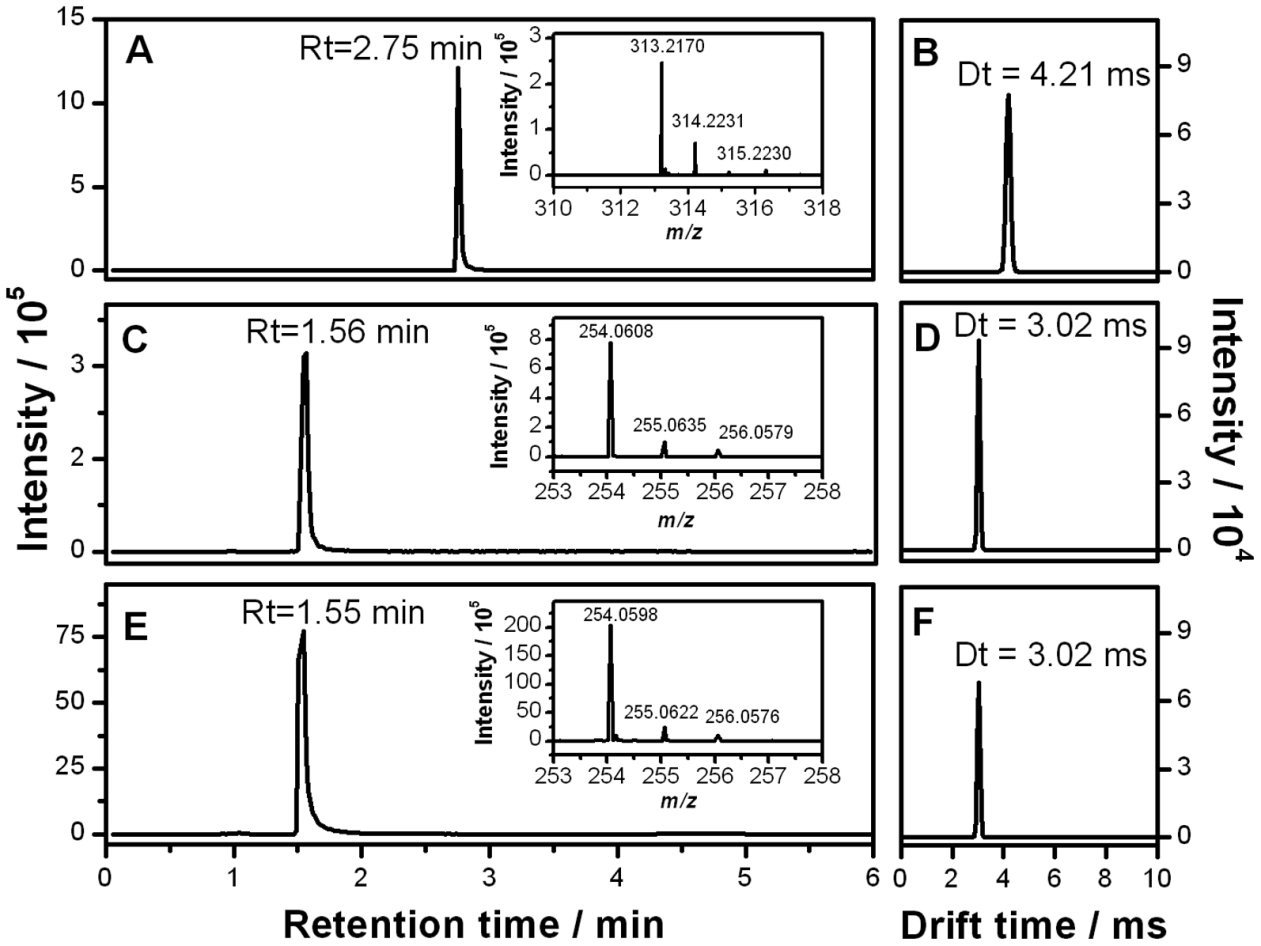
UHPLC-IMS-TOF-MS analysis. Extracted ion chromatogram (A) and extracted ion mobility chronogram (B) for a levonorgestrel standard with *m/z* 313.2168±0.005. Extracted ion chromatogram (C) and extracted ion mobility chronogram (D) for sulfamethoxazole standard with *m/z* 254.0599±0.005. Extracted ion chromatogram (E) and extracted ion mobility chronogram (F) for sulfamethoxazole in the poor quality contraceptive tablet with *m/z* 254.0599±0.005. Mass spectra for the target compounds are displayed as insets.

The most worrisome result was found for sample 2a, with no evidence of levonorgestrel in any of the assays conducted. The lack of API was first suggested by the TLC assay against a levonorgestrel standard and a positive control (Figure 1). This result was confirmed by HPLC-DAD, which also suggested the presence of a different compound in the tablet, eluting at a shorter retention time than levonorgestrel (Figure 2). In order to further investigate the composition of this sample, UHPLC-IMS-MS was conducted. First, a levonorgestrel standard was analyzed to establish the retention time, the drift time and the mass spectral features of the correct API. Figures 3a–b illustrate the extracted ion chromatogram and the extracted ion mobility chronogram for the [levonorgestrel+H]^+^ ion at *m/z* 313.2168 obtained for a 10 µM standard, which exhibited a retention time and drift time of 2.75 min and 4.21 ms, respectively. Under identical conditions, no peak was observed in the extracted ion chromatogram and ion mobility chronogram of sample 2a. Instead, the base peak in the chromatogram of the suspicious sample was given by a compound that eluted at 1.55 min, with a monoisotopic *m/z* of 254.0598. Assuming that the peak of interest in the spectrum corresponded to the [M+H]^+^ ion, elemental formulae were generated based on the expected mass accuracy and experimental isotopic pattern, and the possible candidate molecules were searched against the Metlin database [21]. In this way, the wrong API in sample 2a was identified as the antibiotic sulfamethoxazole and confirmed by UHPLC-IMS-MS analysis of a sulfamethoxazole standard. Figures 3c–d show the extracted ion chromatogram, and the extracted ion mobility chronogram for the [sulfamethoxazole+H]^+^ ion at *m/z* 254.0599 obtained for a 10 µM standard, which exhibited a retention time and drift time of 1.561min and 3.02 ms, respectively. The extracted ion chromatogram, and the extracted ion mobility chronogram for the [sulfamethoxazole+H]^+^ ion in sample 2a, as well as the corresponding mass spectrum for the target compound, are shown in Figures 3e–f. These results highlight the retention time, drift time, and isotopic pattern matches for the sulfamethoxazole standard and the poor quality ECP. The high resolving power of the time-of-flight analyzer combined with the separation provided by the chromatographic method and ion mobility stage allowed the identification of the main active ingredient in sample 2a. The orthogonality between IMS and MS separations increased the peak capacity of MS alone, providing information-rich data that contributed to the identification of the wrong API in the ECP.

**Figure 4:**
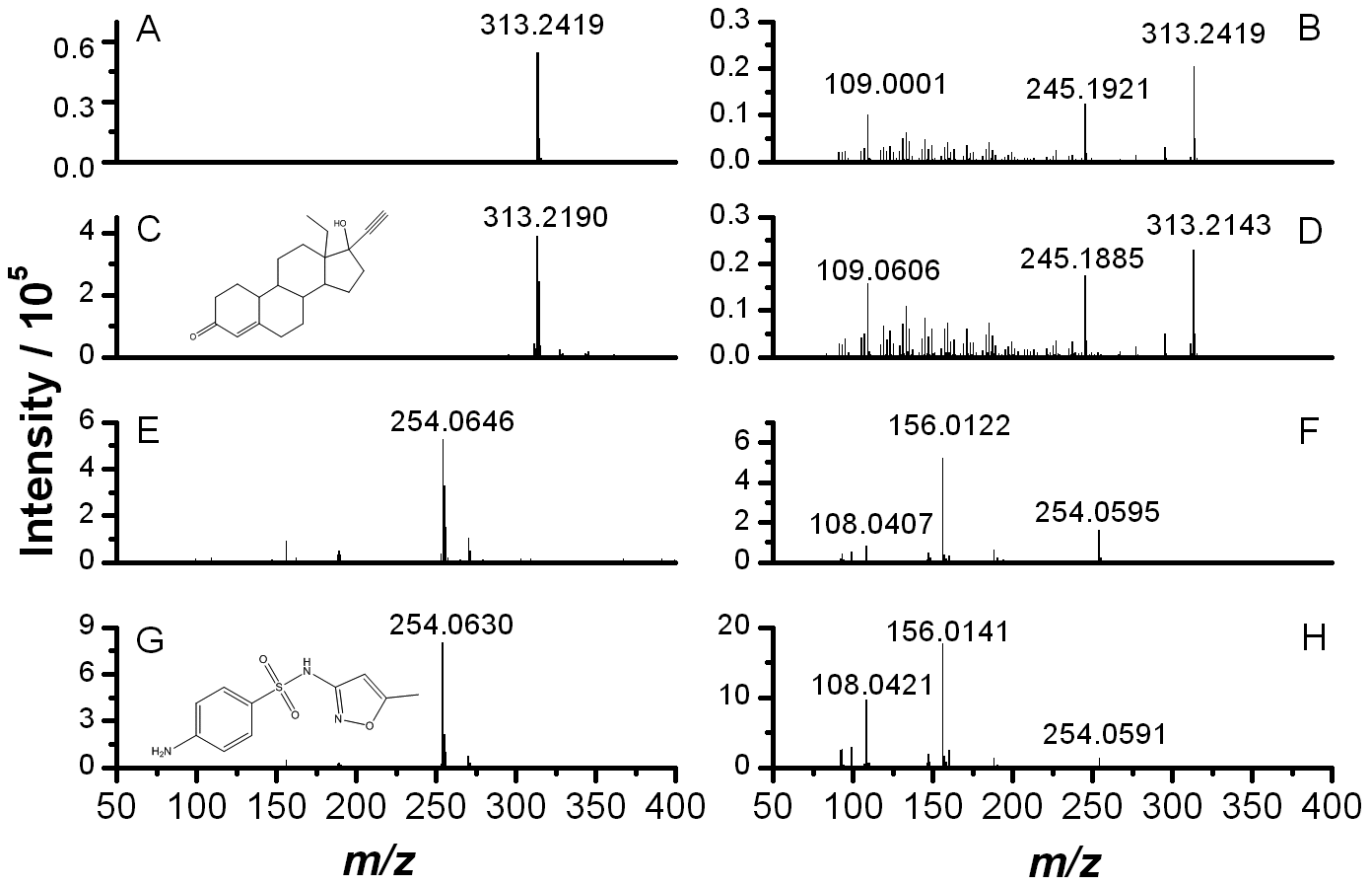
DART MS and MS/MS analysis using a collision energy of 20 eV. (A): Mass spectrum of a contraceptive tablet containing levonorgestrel. (B): MS/MS spectrum for the precursor ion with *m/z* 313.2419 shown in A. (C): Mass spectrum of levonorgestrel standard. (D): MS/MS spectrum for the precursor ion with *m/z* 313.2190 shown in C. (E): Mass spectrum of sample 2a. (F): MS/MS spectrum for the precursor ion with *m/z* 254.0646 shown in E. (G): Mass spectrum of sulfamethoxazole standard. (H): MS/MS spectrum for the precursor ion with *m/z* 254.0630 shown in G.

Further confirmation of the presence of sulfamethoxazole in sample 2a was accomplished by DART-MS and MS/MS, which provides an ionization mechanism which is complementary to those in electrospray ionization [22]. Samples were analyzed with no need of solvents, extractions, processing, or preparation steps by holding a capillary with sample deposited on it in front of the atmospheric pressure inlet of the high resolution mass spectrometer while exposing it to the ionizing plasma. The resulting spectra were obtained in seconds. Figure 4a illustrates the mass spectrum of an authentic levonorgestrel-containing ECP from an authentic sample of the same brand/manufacturer as compared to the packaging for sample 2a except that the tablet strength was 0.75 mg (we were unable to obtain an authentic sample of the authentic 1.5 mg product), which exhibited the expected signal for the [levonorgestrel+H]^+^ ion at *m/z* 313.2419 (Δm = 25.1 mDa). Further fragmentation of this ion provided a unique mass spectral fingerprint of the API (Figure 4b). These mass spectra matched with those obtained for a levonorgestrel standard (Figures 4c and 4d, respectively). In contrast, the mass spectrum of sample 2a exhibited the characteristic monoisotopic peak at *m/z* 254.0646 for the [sulfamethoxazole+H]^+^ ion (Δm = 4.7 mDa), and a completely different fingerprint was obtained from the product ion mass spectrum of that precursor ion (Figures 4e and 4f). Both isotopic and fragmentation patterns were identical to those obtained for a sulfamethoxazole standard (Figures 4g and 4h), further confirming the identity of the ingredient that substituted levornorgestrel in sample 2a. This ambient MS technique provided additional proof of the presence of sulfamethoxazole through a fast and simple approach. Despite the different chemical nature of levonorgestrel and sulfamethoxazole, both compounds were easily ionized by both atmospheric pressure chemical ionization and electrospray ionization mechanisms prevailing in DART-MS and UHPLC-MS, respectively.

Once the identity of the wrong active ingredient in sample 2a was established beyond doubt, the concentration of sulfamethoxazole was determined by UHPLC-QqQ-MS/MS. Triple quadrupole mass spectrometers operated in multiple reaction monitoring (MRM) mode minimize matrix interferences and chemical noise, making it a highly sensitive approach for targeted quantitation. Optimized MRM parameters utilized for quantitation are listed in Table 2. The 254→92 precursor-to-product ion transition was used for sulfamethoxazole quantitation as it provided the highest sensitivity; while the 254→156 transition was used for identity confirmation. A six point external calibration curve (R^2^ = 0.998) was used to determine sulfamethoxazole concentration in sample 2a. The sulfamethoxazole quantity per tablet was determined to be 26±2 mg, equivalent to 19±1% w/w.

**Table 2:** Precursor ion, fragmentor voltage, collision cell voltage, and product ions for sulfamethoxazole MRM analysis.

Analyte	Precursor Ions (*m/z*)	Fragmentor Voltage/V	Collision cell Voltage/V	Product Ions (*m/z*)
**Sulfamethoxazole [M+H]^+^**	254	122	28	92^Q^
	254	122	12	156^C^

Q Quantifying ion; ^C^ Confirming ion.

**Figure 5:**
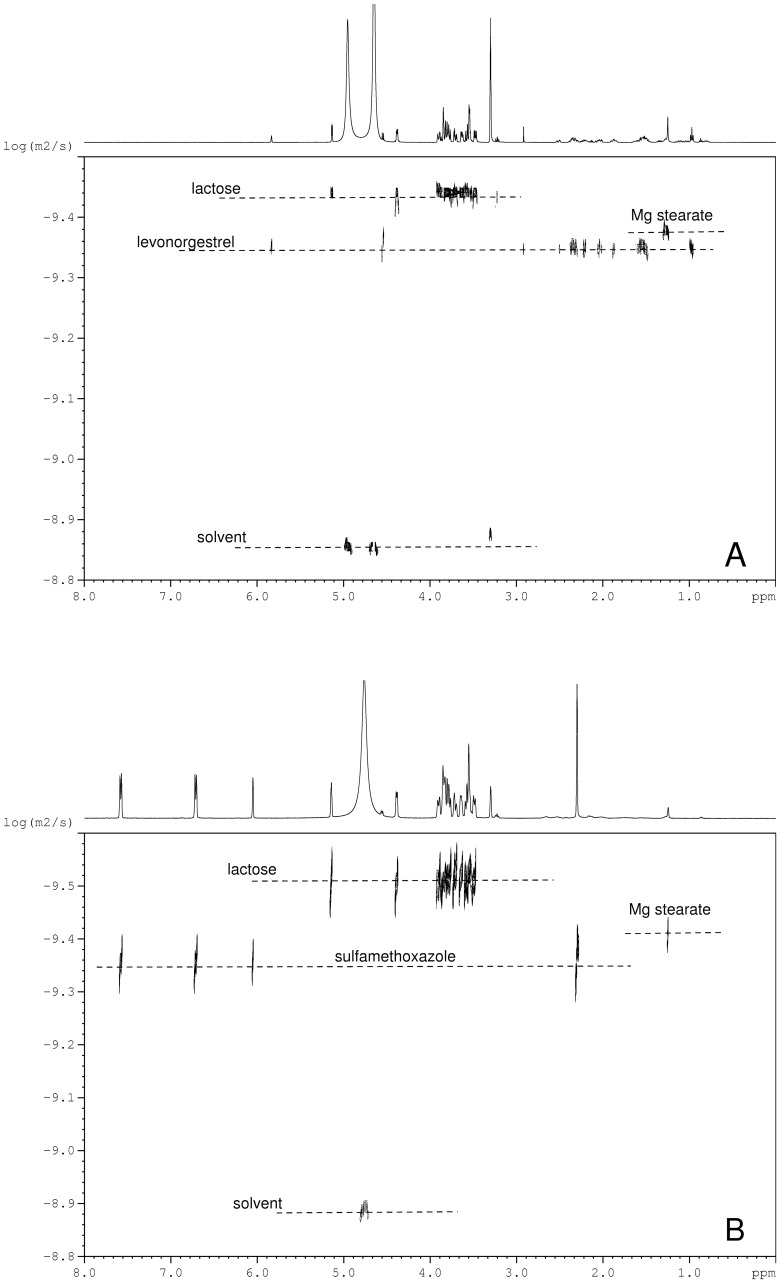
2D DOSY ^1^H NMR analysis. Spectra (log D vs. chemical shift) for levonorgestrel-containing (A) and sample 2a (B) contraceptive tablets dissolved in CD_4_O:D_2_O (80∶20 v/v). Signals observed at 3.3 ppm and in the range 4.6–5 ppm are due to the deuterated solvent mixture.

Further analysis was performed via 2D DOSY ^1^H NMR spectroscopy to investigate the identity of excipients present in sample 2a and compare those to what was present in authentic samples with levonorgestrel from the same stated manufacturer. 2D DOSY ^1^H NMR has been successfully utilized in combination with DART-MS for the investigation of falsified antimalarials [23]. Figure 5 shows the 2D DOSY ^1^H NMR spectra for the samples dissolved in CD_4_O:D_2_O 80∶20 v/v. Both samples presented two soluble excipients that were identified as lactose (*D*∼316 µm s^−1^) and magnesium stearate (*D*∼407 µm s^−1^); which are typically found in pharmaceutical formulations [24]. Characteristic signals in the ^1^H NMR spectrum of lactose included two doublets at 4.38 and 4.55 ppm associated with the protons of the disaccharide anomeric carbons; a triplet at 3.22 ppm characteristic of the proton bonded to the carbon involved in the glycosidic linkage in the glucose moiety; and several signals between 3.48 and 3.92 ppm for the hydroxyls and remaining protons. Magnesium stearate was evidenced by a main peak at 1.25 ppm, and signals at 0.87, 1.55 and 2.13 ppm. The confirmation of the identity of these compounds was further assessed by comparison with standards (Figures S1–S2). Although the principal ingredients exhibited similar diffusion coefficients in both samples, the ^1^H NMR spectra provided very different spectroscopic fingerprints both in terms of chemical shifts and couplings characteristic of levonorgestrel (*D*∼436 µm s^−1^) or sulfamethoxazole (*D*∼446 µm s^−1^). Characteristic signals of sulfamethoxazole included two singlets at 2.3 and 6.05 ppm, and two doublets at 6.71 and 7.59 ppm, which clearly do not overlap with any signal associated with levonorgestrel (Figures S1–S3). In all spectra signals observed at 3.3 ppm and in the range 4.6–5 ppm were due to the deuterated solvent mixture. These experiments validated previous MS results by confirming the presence of sulfamethoxazole in sample 2a, and provided a further characterization of the sample in terms of excipients. In this way, the analytical strategy based on different complimentary techniques delivered a clear fingerprint of the composition of this poor quality ECP.

## Discussion

Levonorgestrel tablets for emergency contraception are currently available in two dosage forms, either one tablet of 1.5 mg or two tablets each of 0.75 mg. Label instructions advise to use the medication within 72 hours of unprotected intercourse, with the second tablet taken 12 hours after the first one when using the two-0.75 mg tablet dosage form. Whereas the 72-hour period is the recommended timeframe for use, some evidence does suggest that increased effectiveness is achieved when taken as soon as possible, with moderate effectiveness provided from 72 to 120 hours after coitus [16], [25]. When taken as instructed, levonorgestrel tablets can reduce the risk of pregnancy by approximately 80% (per “plan B” label, for example) relative to the number of pregnancies that would be expected to occur with unprotected intercourse [16], [25], [26]. This product type is only effective when taken before ovulation [27], [28].

Assuming that the *in vitro* dissolution method predicts the expected *in vivo* release rate of the API, sample 10 may lead to little to no contraceptive action due to the lack of levonorgestrel release. Furthermore, the lower levels of levonorgestrel release for samples 2c, 3b, 4, and 6, with the wide range of tablet content found in sample 17, suggest that these particular product batches may not yield the full level of contraceptive effectiveness expected, especially considering the suggested importance of taking the medication in a timely manner after intercourse but before ovulation [16], [25], [26]. Only 72% of the batches analyzed in the present market survey would be expected to provide the stated level of contraceptive action. Of the remaining batches, 20% would be expected to provide questionable results (samples 2c, 3b, 4, 6, 17), with 8% offering little to no prevention against unwanted pregnancy (samples 2a and 10).

Sample 2a would present the highest risk to the consumer due to the lack of expected contraceptive action and the potential complications that may arise from the presence of sulfamethoxazole. Sulphonamide antibiotics have been found before as wrong active ingredients in falsified medicines, as is the case of sulfamethoxazole and sulfadoxine in falsified artesunate [29]. Sulphonamides have a wide range of adverse effects, such as rash and Stevens-Johnson syndrome, and may also interact with other commonly-used medicines. Without the patient's or health workers awareness of sulpha medicine consumption, any adverse effects or interactions would be clinically very confusing.

Following the identification of sulfamethoxazole in sample 2a, the Peruvian medicine regulatory authority, DIGEMID (Dirección General de Medicamentos Insumos y Drogas), was informed [30], [31]. Additionally, the manufacturer indicated on the packaging for sample 2a was contacted regarding these results. The manufacturer indicated that their facilities dedicated to the production of hormonal contraceptives are separated from the facilities used in the manufacturing of other pharmaceuticals, thus preventing the risk of contamination. The manufacturer also stated that a local distributor in Peru had reported to DIGEMID that the entire stock of the original sample of 2a had been stolen, thus raising suspicion that sample 2a is fraudulent. Furthermore, the distributor had also alerted DIGEMID about fraudulent samples of product 2b on the market, providing genuine and fraudulent samples of 2b to DIGEMID for further investigation. Although we obtained what apparently is a genuine sample of 2b, the results obtained for 2c are leading the manufacturer to investigate the possibility of fraudulent 2c product.

The detection and comprehensive characterization of falsified pharmaceuticals can provide critical information that contributes to avoid severe consequences to the patient. Here, we have proven that the combination of two different mass spectrometric-based methods that utilized chromatographic and ion mobility separations, and ambient ionization techniques allowed the identification of the unknown wrong API in the batch suspected to be fraudulent, and a third mass spectrometric-based method provided successful quantitation of the drug in the poor quality ECP. As well, qualitative characterization in terms of excipients was accomplished by means of 2D DOSY 1H NMR spectroscopy. Therefore, a multi-platform analytical strategy based on sophisticated complimentary techniques can provide exhaustive characterization of falsified medicines and becomes crucial in the screening of poor quality medicines.

Overall, these results highlight the diversity of drug quality problems that have important implications for public health, and especially women's health and society. These ranged from inadequate amount and release of levonorgestrel to the presence of a wrong active ingredient. This work also emphasizes the need for efficient oversight of pharmaceutical products, with proper monitoring of manufacturers and distribution mechanisms (with proper storage and supply chain security) in order to lower the risk of users being exposed to products of poor quality, safety, and efficacy. The fact that falsified emergency contraception has been reported in Nigeria, Ghana, Kenya, Angola and USA [15] and now South America is also known to be afflicted, suggests that it is an important and widespread public health problem. The WHO has been conducting a prequalification program for pharmaceuticals which historically has placed a significant emphasis on HIV, malaria and tuberculosis medications, with growing effort for reproductive health products [32]. Currently there are three products WHO prequalified for emergency contraception. Hopefully, resources will be allocated for additional prequalification of ECPs to provide further assistance for improving and assuring product quality in the global market for this important class of products. The fact that even pre-qualified emergency contraception products have been targeted by counterfeiters in Africa [15] strongly suggests that more resources should be invested in checking the medicine supply.

## Conclusions

The chemical quality of emergency contraceptives (0.75 mg and 1.5 mg levonorgestrel tablets) in Lima, Peru, was evaluated. Of the 25 different batches analyzed, 7 batches were non-complaint with product specifications; mainly due to inadequate levonorgestrel dissolution. The most striking result was provided by a batch that contained a wrong active ingredient with no levonorgestrel detected. Mass spectrometry and 2D DOSY ^1^H NMR spectroscopy identified the unknown compound as the antibiotic sulfamethoxazole. Government authorities in Peru and the stated manufacturer were alerted of the findings for further investigation of the source of this product.

## Supporting Information

Figure S1 **^1^H NMR spectra.** a) genuine ECP, b) levonorgestrel standard; c) lactose standard; d) Mg stearate standard dissolved in CD_4_O:D_2_O (80∶20 v/v). Signals observed at 3.3 ppm and in the range 4.6–5 ppm are due to the solvent.
(TIF)

Figure S2 **^1^H NMR spectra.** a) sample 2a, b) sulfamethoxazole standard; c) lactose standard; d) Mg stearate standard dissolved in CD_4_O:D_2_O (80∶20 v/v). Signals observed at 3.3 ppm and in the range 4.6–5 ppm are due to the solvent.
(TIF)

Figure S3 **^1^H NMR spectra.** Genuine (top) and poor quality (bottom) contraceptive tablet dissolved in CD_4_O:D_2_O (80∶20 v/v). Signals observed at 3.3 ppm and in the range 4.6–5 ppm are due to the solvent.
(TIF)
