# Supplementary figures and images for: A Tiered Analytical Approach for Investigating Poor Quality Emergency Contraceptives
Source: PLoS One. 2014 Apr 18;9(4):e95353. doi: 10.1371/journal.pone.0095353 (PMC3991657; doi:10.1371/journal.pone.0095353)

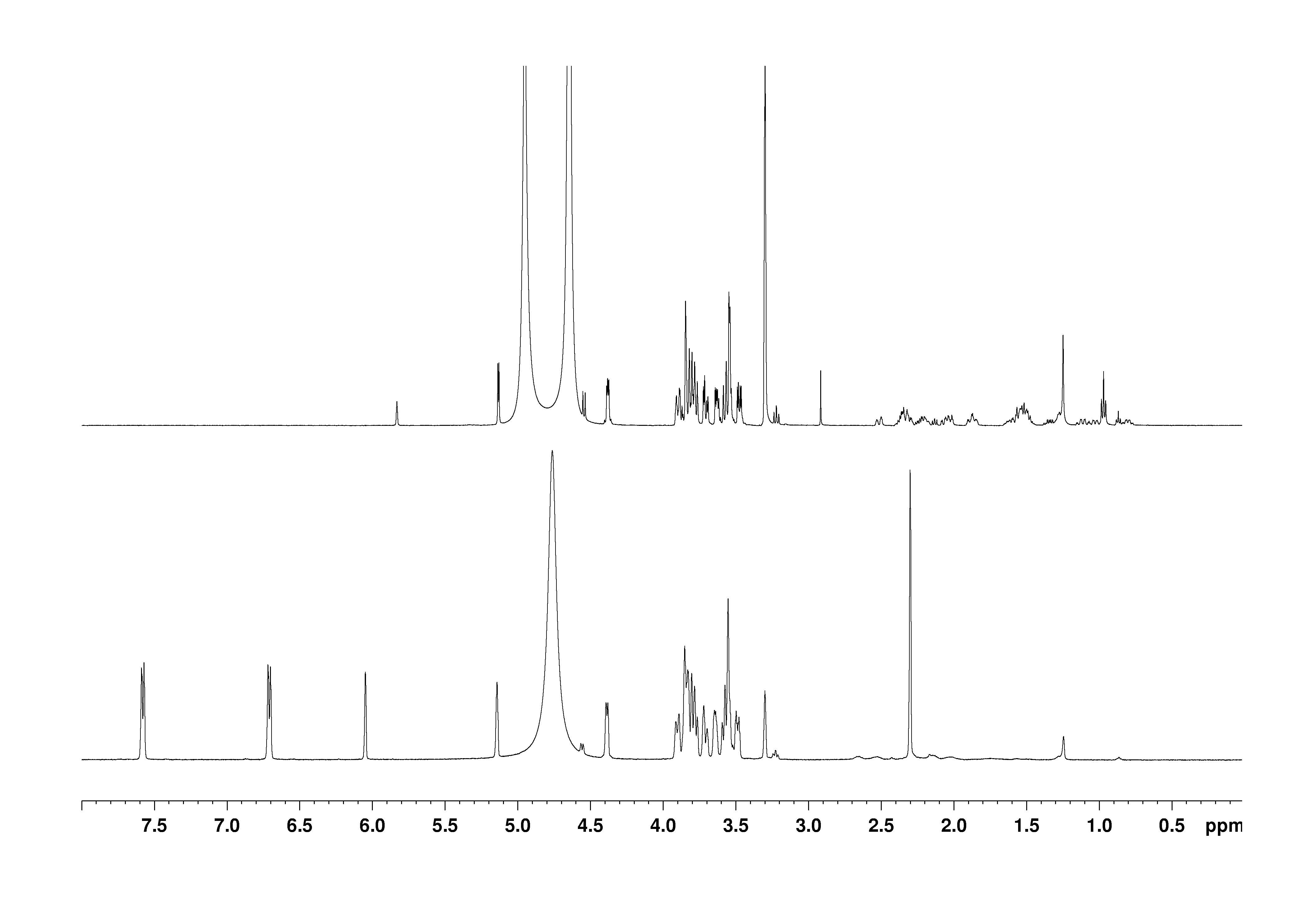

Supplement: Figure S3 — 1H NMR spectra. Genuine (top) and poor quality (bottom) contraceptive tablet dissolved in CD4O:D2O (80∶20 v/v). Signals observed at 3.3 ppm and in the range 4.6–5 ppm are due to the solvent. (TIF) [file pone.0095353.s003.tif]
